# Fully automated MR-based virtual biopsy of primary CNS lymphomas

**DOI:** 10.1093/noajnl/vdae022

**Published:** 2024-03-14

**Authors:** Vicky Parmar, Johannes Haubold, Luca Salhöfer, Mathias Meetschen, Karsten Wrede, Martin Glas, Maja Guberina, Tobias Blau, Denise Bos, Anisa Kureishi, René Hosch, Felix Nensa, Michael Forsting, Cornelius Deuschl, Lale Umutlu

**Affiliations:** Department of Diagnostic and Interventional Radiology and Neuroradiology, University Hospital Essen, Essen, Germany; Institute for Artificial Intelligence in Medicine, University Hospital Essen, Essen, Germany; Department of Diagnostic and Interventional Radiology and Neuroradiology, University Hospital Essen, Essen, Germany; Institute for Artificial Intelligence in Medicine, University Hospital Essen, Essen, Germany; Department of Diagnostic and Interventional Radiology and Neuroradiology, University Hospital Essen, Essen, Germany; Institute for Artificial Intelligence in Medicine, University Hospital Essen, Essen, Germany; Department of Diagnostic and Interventional Radiology and Neuroradiology, University Hospital Essen, Essen, Germany; Institute for Artificial Intelligence in Medicine, University Hospital Essen, Essen, Germany; Department of Neurosurgery and Spine Surgery, University Hospital Essen, Essen, Germany; Department of Neuropathology, University Hospital Essen, Essen, Germany; Department of Radiotherapy, University Hospital Essen, Essen, Germany; Department of Neurology and Neurooncology, University Hospital Essen, Essen, Germany; Department of Diagnostic and Interventional Radiology and Neuroradiology, University Hospital Essen, Essen, Germany; Institute for Artificial Intelligence in Medicine, University Hospital Essen, Essen, Germany; Department of Diagnostic and Interventional Radiology and Neuroradiology, University Hospital Essen, Essen, Germany; Institute for Artificial Intelligence in Medicine, University Hospital Essen, Essen, Germany; Department of Diagnostic and Interventional Radiology and Neuroradiology, University Hospital Essen, Essen, Germany; Institute for Artificial Intelligence in Medicine, University Hospital Essen, Essen, Germany; Department of Diagnostic and Interventional Radiology and Neuroradiology, University Hospital Essen, Essen, Germany; Department of Diagnostic and Interventional Radiology and Neuroradiology, University Hospital Essen, Essen, Germany; Department of Diagnostic and Interventional Radiology and Neuroradiology, University Hospital Essen, Essen, Germany

**Keywords:** artificial intelligence, central nervous system, glioma, lymphoma, neoplasms magnetic resonance imaging

## Abstract

**Background:**

Primary central nervous system lymphomas (PCNSL) pose a challenge as they may mimic gliomas on magnetic resonance imaging (MRI) imaging, compelling precise differentiation for appropriate treatment. This study focuses on developing an automated MRI-based workflow to distinguish between PCNSL and gliomas.

**Methods:**

MRI examinations of 240 therapy-naive patients (141 males and 99 females, mean age: 55.16 years) with cerebral gliomas and PCNSLs (216 gliomas and 24 PCNSLs), each comprising a non-contrast T1-weighted, fluid-attenuated inversion recovery (FLAIR), and contrast-enhanced T1-weighted sequence were included in the study. HD-GLIO, a pre-trained segmentation network, was used to generate segmentations automatically. To validate the segmentation efficiency, 237 manual segmentations were prepared (213 gliomas and 24 PCNSLs). Subsequently, radiomics features were extracted following feature selection and training of an XGBoost algorithm for classification.

**Results:**

The segmentation models for gliomas and PCNSLs achieved a mean Sørensen–Dice coefficient of 0.82 and 0.80 for whole tumors, respectively. Three classification models were developed in this study to differentiate gliomas from PCNSLs. The first model differentiated PCNSLs from gliomas, with an area under the curve (AUC) of 0.99 (F1-score: 0.75). The second model discriminated between high-grade gliomas and PCNSLs with an AUC of 0.91 (F1-score: 0.6), and the third model differentiated between low-grade gliomas and PCNSLs with an AUC of 0.95 (F1-score: 0.89).

**Conclusions:**

This study serves as a pilot investigation presenting an automated virtual biopsy workflow that distinguishes PCNSLs from cerebral gliomas. Prior to clinical use, it is necessary to validate the results in a prospective multicenter setting with a larger number of PCNSL patients.

**Key Points**

- The proposed workflow separates PCNSL and gliomas with an AUC of 0.99 (sensitivity: 0.6, specificity: 1.0).
- This pilot study distinguishes high- and low-grade gliomas from PCNSL using machine learning.
- Automatic segmentations show a mean overlap with the manual equals of 81% (Dice coefficient of 0.81).

**Importance of the Study**

Primary central nervous system (CNS) lymphoma manifests as the proliferation of malignant cells within the lymphatic tissue located in the cerebral and spinal regions. According to current treatment recommendations, high-grade gliomas are typically treated with an aggressive resection, while PCNSLs are usually treated with systemic therapy. Given the potential radiographic similarity between PCNSLs and cerebral gliomas on MRI, separating the two is of utmost significance to facilitate a precise treatment regime. The current study illustrates an automated workflow to precisely distinguish between PCNSLs and gliomas by using radiomics features extracted from MRI images of the patients and the automatically generated tumor segmentations.

In their study, Gillies et al.^1^ concluded that images are not just a visual tool; they represent data. The process of acquiring or extracting numerous quantitative features from tomographic images (computed tomography [CT], magnetic resonance imaging [MRI], or positron emission tomography [PET]) is known as radiomics.^1^ The role of feature-based radiomics and deep-learning-based radiomics is increasingly evaluated in neuro-oncology.^2^ Radiomics features can be extracted from the tumor segmentations and used to train machine-learning models.^3–7^ Haubold et al.^3^ showed that the genetic profile and grading of cerebral gliomas could be predicted in a fully automated manner from day-to-day business MR scans.

Primary central nervous system lymphomas (PCNSLs) are considered one of the deadliest types of neoplasms. Once a rare intracranial neoplasm, the incidence of PCNSLs has substantially increased in the last decades.^8^ Though PCNSLs have some characteristic MRI findings, they might overlap with other intracerebral neoplasms.^9^ Sometimes, their appearance in MR imaging can be deceiving to the eyes, and it is not easy to differentiate PCNSLs from gliomas.^9,10^

Therefore, the precise differentiation of PCNSL from gliomas is the key to finding the correct treatment regime for the patient.^11–13^ The current treatment guidelines adopt systemic therapy, including chemotherapy, targeted therapies, or whole-brain radiotherapy for PCNSL, while for high-grade gliomas, an aggressive resection is often recommended.^14,15^

Apart from the differentiation of cerebral gliomas, a tissue sampling is crucial for the treatment of a PCNSL due to various reasons, including the extended and toxic nature of the high-dose methotrexate-based treatment, especially in elderly patients.^16^ Additionally, the growing significance of molecular markers in new treatment options^17^ and the difficulty in acquiring this information after the start of treatment and achievement of remission—a time when it becomes unattainable unless there is a recurrence of the lymphoma—further emphasize its importance.

Previous studies have shown multiple ways to extract quantitative information from images, i.e. a radiomics-based approach or a deep-learning-based feature extraction, to decode different types of tumors. In this regard, Kim et al.^6^ and Chen et al.^18^ concluded that radiomics features derived from multi-parametric MRI could effectively differentiate PCNSLs from glioblastomas.

Current studies neglect differential diagnosis and focus on differentiating a type of glioma (typically high-grade glioma or glioblastoma) from PCNSL.^3,6,7,18–23^ Furthermore, those mentioned above fully automated approaches to distinguish different mutations of gliomas^3^ cannot be used clinically if diagnoses like PCNSLs are not determined. Based on that, this study aims to develop a fully automated approach that utilizes a simple MRI protocol to differentiate PCNSL from cerebral gliomas.

## Material and Methodology

### Ethics Statement

This study was performed in adherence to all guidelines defined by the approving institutional review board of the investigating hospital (approval code 21-10487-BO). The Institutional Review Board waived written informed consent due to the study's retrospective nature. Complete anonymization of all data was performed before inclusion in the study.

### Study Design and Cohort

MRI examinations of a total of 240 therapy-naive patients from 2013 to 2020 (141 males and 99 females with a mean age of 55.16 ± 17.06 years) with cerebral gliomas and PCNSLs (gliomas: *n* = 216; PCNSL: *n* = 24) were included in the study. Patients with previous brain surgery, pretreatments of the brain tumor, lack of histopathology, or incomplete MRI examinations were excluded from the analysis. The imaging protocol comprised a FLAIR sequence, a non-contrast T1-weighted, and a contrast-enhanced T1-weighted sequence. The study cohort encompassed 186 high-grade gliomas (classified as per the WHO III and WHO IV criteria) and 30 low-grade gliomas (classified as per the WHO I and WHO II criteria). Supplementary Appendix F for reference delineates a comprehensive genetic distribution within the cohort. Notably, all participants presented with primary central nervous system lymphoma (PCNSL), and individuals with HIV-associated PCNSL were deliberately excluded from the study. It is pertinent to note that none of the patients within the cohort were administered steroids prior to the radiological assessment.

**Figure 1.**
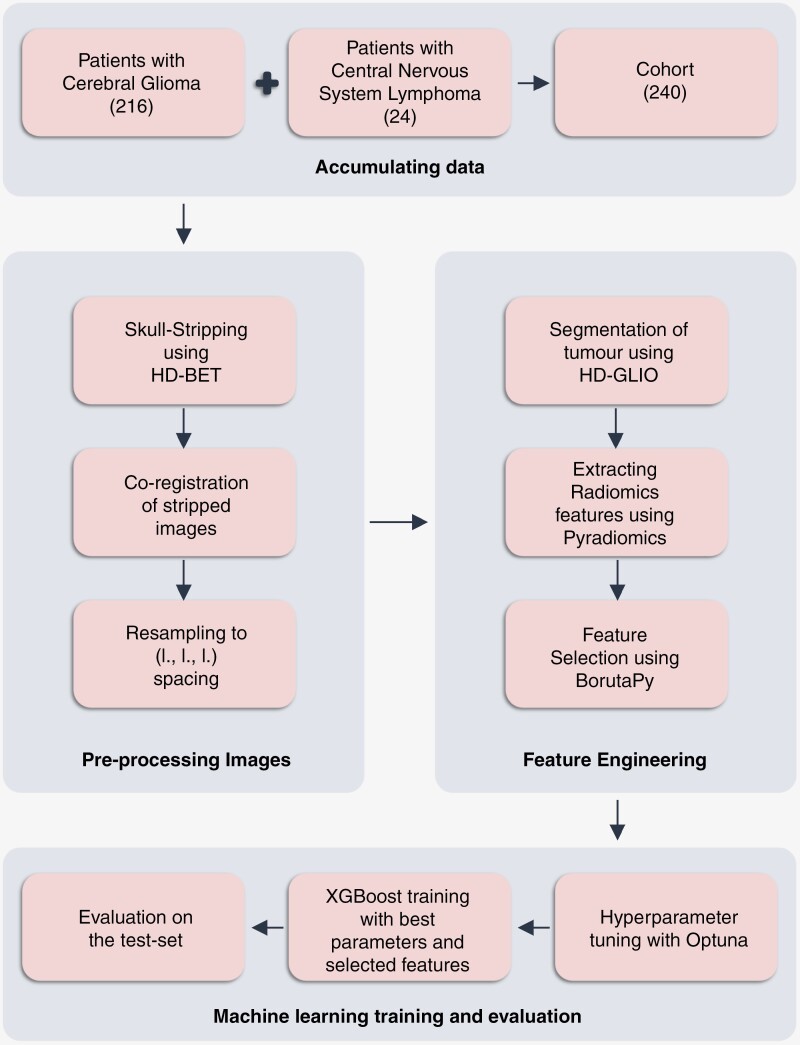
Automated virtual biopsy workflow in differentiating PCNSLs from gliomas.

The training set contained 192 patients (173 with glioma and 19 with PCNSL), and the test set comprised 48 patients (43 with glioma and 5 with PCNSL). All images were anonymized and co-registered after skull-stripping. After that, the images were resampled to a standard spacing of [1.0, 1.0, 1.0] to ensure compatibility with the input images on which the segmentation model was trained. Segmentations were generated using HD-GLIO^24,25^ trained on 3220 MRI examinations from 1450 brain tumor patients (80% for training and 20% for testing). HD-GLIO underwent training through the utilization of ground-truth tumor segmentation masks and a set of pre-contrast T1-weighted, contrast-enhanced T1-weighted, T2-weighted, and FLAIR sequences, all of which were subject to brain extraction and co-registration procedures. Because of the lack of availability of T2-weighted images in the analyzed data, FLAIR imaging was used as input for 2 channels. The segmentation efficiency was then assessed for potential impact due to this change of input sequence. Therefore, 213 glioma patients and 24 PCNSL patients were manually segmented by 2 radiology residents and reviewed and corrected in consensus by 2 radiology consultants. Radiomics features were extracted from the segmentations and given as inputs to the machine learning model to differentiate PCNSLs from gliomas. The automated pipeline, i.e. the workflow for automated virtual biopsy, is depicted in Figure 1.

### MR Imaging

At a single center, various 1.5T and 3T MR machines MAGNETOM Symphony, MAGNETOM Sonata, MAGNETOM Avanto, MAGNETOM Aera, MAGNETOM Skyra, MAGNETOM Espree, and Biograph mMR from a single vendor (Siemens Healthineers AG, Erlangen, Germany) were used to conduct the MRI exams. The training dataset comprised 118 MRI examinations conducted on a 1.5 T scanner and 71 examinations conducted on a 3T scanner. In contrast, the testing dataset consisted of 33 scans from a 1.5 T MRI scanner and 14 from a 3T MRI scanner. The distribution of studies taken from different scanners and their properties are shown in Supplementary Tables Appendix A—Appendix D.

### Brain Extraction

Brain extraction is an integral part of preprocessing. It ensures anonymity and removes any unnecessary structures from the images. It was done using the HD-BET extraction tool.^26^ The masks of the extracted brain parenchyma were saved and applied to the images after co-registration.

### Co-Registration

FLAIR and non-contrast T1-weighted extracted brain sequences were co-registered to spatially align with contrast-enhanced T1-weighted datasets. The transform matrices for this registration were saved and then applied to the non-stripped (not brain-extracted) images. The co-registration was performed using ANTsPy (Advanced Normalization Tools in Python). ANTsPy wraps ANTs (Advanced Normalization Tools) (a C++ biomedical image processing library) along with the statistical capabilities of ANTsR (Advanced Normalization Tools in R) to seamlessly integrate numpy, scikit-learn, and the more excellent Python community.^27–29^ A rigid registration, i.e. translation and rotation, was performed during the co-registration.^30^ The co-registration was first performed on the stripped images, and then subsequently, the transform matrix was used to perform co-registration on the original non-stripped images.

### Segmentation

**Figure 2.**
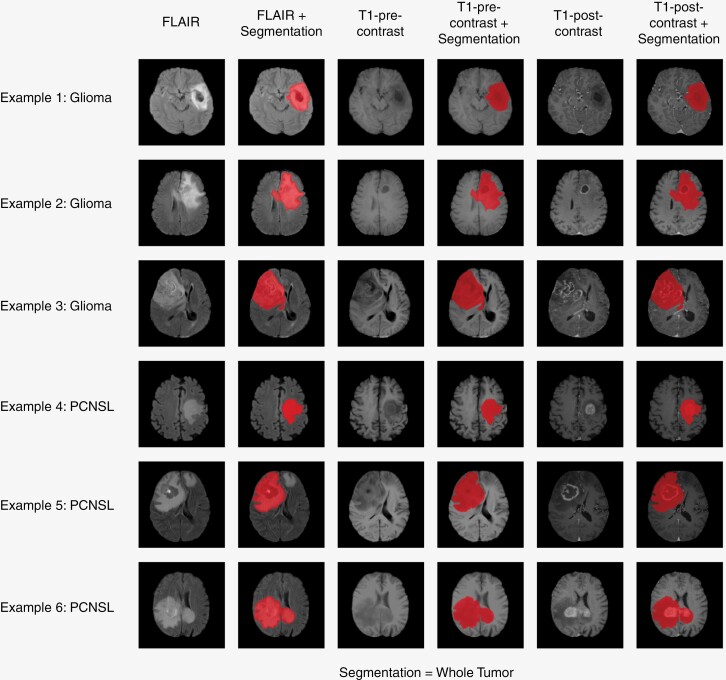
An assemblage featuring instances of tumor segmentations produced by the HD-GLIO network is presented. The composition comprises 3 illustrative examples for each class—namely, gliomas and PCNSLs. Each exemplar encompasses slices extracted from 3 MRI series utilized for segmentation generation: FLAIR, pre-contrast T1-weighted, and post-contrast T1-weighted images, accompanied by a segmentation overlay for each respective series.

The segmentations were automatically generated using HD-GLIO. HD-GLIO utilizes a nnU-Net.^24^ The network was trained on FLAIR, non-contrast T1-weighted, T2-weighted, and contrast-enhanced T1-weighted datasets. HD-GLIO was trained on 3220 MRI examinations from 1450 brain tumor patients (80% for training and 20% for testing) with a slice spacing of (1.0, 1.0, 1.0) mm^3^. The study cohort was, therefore, resampled to the same spacing. The study cohort did not contain T2-weighted images; hence, FLAIR imaging was enrolled twice as input. Because of this change, the glioma segmentation was validated on 213 manual glioma segmentations, and the PCNSL segmentations were validated using 24 manual PCNSL segmentations. Figure 2 illustrates segmentations generated by HD-GLIO.

### Radiomics Feature Extraction

Once the segmentations were generated, radiomics features were extracted using PyRadiomics.^31,32^ The extracted features consisted of first-order statistics features, shape-based features, Gray Level Co-Occurrence Matrix (GLCM) features, Gray Level Run Length Matrix (GLRLM) features, Gray Level Size Zone Matrix (GLSZM) features, Neighboring Gray Tone Difference Matrix (NGTDM) features and Gray Level Dependence Matrix (GLDM) features. Relevant features were extracted from filter-transformed images using Wavelet transformation, Laplacian of Gaussian (LoG) transformation, Local Binary Pattern 3D (LBP3D) transformation, and Gradient transformation.

### Feature Selection

Training a model with more features frequently results in overfitting, where the model excels on the training data but underperforms on unseen data. The selection of relevant features diminishes the risk of overfitting, enhancing generalizability and reducing the likelihood of capturing data noise. BorutaPy,^33^ an implementation of the Boruta algorithm in Python, was used for feature selection to reduce the noise due to redundant or similar features. Boruta represents an all-relevant feature selection approach, unlike many others focusing on minimal optimality. It aims to identify all features containing valuable information for prediction rather than seeking a potentially concise subset of features where a classifier minimally errors.^33,34^ BorutaPy requires a supervised learning estimator, and hence, the XGBoost algorithm^35^ was passed on as an estimator to BorutaPy for the optimal feature set. Thirty-one features were selected for the model for classifying the tumor as PCNSL or glioma. In contrast, 34 features were chosen for differentiating high-grade gliomas from PCNSL and 15 for differentiating low-grade gliomas from PCNSL.

### Training

A simple XGBoost model was used to classify whether the given study or case was diagnosed as PCNSL or glioma. The XGBoost algorithm was trained on the selected features to differentiate PCNSLs from gliomas. Optuna was used on the train set to find the optimal hyperparameters for the XGBoost model. Optuna is an automatic hyperparameter optimization software framework for deep learning and machine learning.^36,37^ Furthermore, each iteration included cross-validation to maximize the stability of the model. The final model was trained on the optimal hyperparameters selected from Optuna. This process was repeated for all 3 models: one differentiating all gliomas from PCNSLs, the other distinguishing low-grade gliomas from PCNSLs, and the last one decoding the tumor as either a high-grade glioma or PCNSLs. Table 1 provides a list of hyperparameters used for training the models. To encounter the imbalanced nature of the dataset, the *scale_pos_weight* parameter was used during training.

**Table 1.** Hyperparameters used to train the models for distinguishing gliomas from PCNSLs

Parameter	Model for glioma vs. PCNSL	Model for high-grade glioma (HGG) vs. PCNSL	Model for low-grade glioma (LGG) vs. PCNSL
booster	dart	dart	dart
gamma	5.91 e−8	0.01	0.091
grow_policy	lossguide	depthwise	depthwise
max_depth	4	5	9
n_estimators	100	100	100
scale_pos_weight	9.11	7.74	1.32
alpha	5.34 e−6	7.64e−6	2.64e−7
eta	0.59	0.42	0.51
normalize_type	forest	forest	forest
sample_type	weighted	uniform	uniform
rate_drop	0.0033	0.061	0.091
skip_drop	0.0005	0.016	0.005
learning_rate	0.3	0.01	0.3
random_state	0	24	24

### Technical Project-Related Terminologies and Their Descriptions

#### BorutaPy.—

BorutaPy is an implementation of the Boruta algorithm in Python. It is a widely used feature selection method that selects all relevant features pertaining to the outcome of the classifier.^33,34^

#### HD-BET.—

HD-BET is an automated brain extraction tool developed as a joint project between the Department of Neuroradiology at the Heidelberg University Hospital and the Division of Medical Image Computing at the German Cancer Research Center (DKFZ).^26^

#### HD-GLIO.—

HD-GLIO is a brain tumor segmentation tool. It was a joint project between the Department of Neuroradiology at the Heidelberg University Hospital, Germany and the Division of Medical Image Computing at the German Cancer Research Center (DKFZ) Heidelberg, Germany.^24,25^

#### Sørensen–Dice coefficient.—

The Dice similarity coefficient, also known as the Sørensen–Dice index or simply Dice coefficient, is a statistical tool which measures the similarity between two sets of data.^38^

#### XGBoost.—

XGBoost is a scalable machine learning system for tree boosting algorithm.^35^

For additional references please see Appendix E.

## Results

### Validation of Glioma and PCNSL Segmentation Models

The segmentation models described in this section aim to accurately delineate gliomas and PCNSLs in MR images. To evaluate the performance of these models, they were validated on datasets of manually segmented images.

Validation was performed on 213 manually segmented glioma studies to evaluate the segmentation efficiency of cerebral gliomas. The Sørensen–Dice coefficient is a standard metric for assessing the performance of image segmentation algorithms. It measures the overlap between the predicted and ground-truth segmentation, with a score 1 indicating perfect overlap and 0 indicating no overlap. In this case, the mean Sørensen–Dice coefficient for segmenting cerebral gliomas was 0.82 ± 0.13. This suggests that the model could accurately segment the gliomas in the images, with a mean overlap of approximately 82% with the ground-truth segmentations.

Similarly, the segmentation efficiency of PCNSLs was validated on 24 manually segmented PCNSL datasets. The mean Sørensen–Dice coefficient for this validation was 0.80 ± 0.19. This indicates that the model could accurately segment the lymphomas in the images, with a mean overlap of approximately 80% with the ground-truth segmentations.

### Dummy Classifiers

Three dummy classifiers were developed to account for the imbalanced class distribution in the dataset (i.e. there are significantly more gliomas than PCNSLs). A dummy classifier is a simple model used as a baseline for comparison. The performance of this model was evaluated using the area under the curve (AUC) metric, which measures the ability of the model to distinguish between positive and negative samples.

The first dummy classifier was designed to predict that any given sample is always a glioma. The second classifier considered the class stratification in the train set, and the last generated predictions uniformly at random from the list of unique classes observed in the dataset, i.e. each type has equal probability. Table 2 shows the results of these classifiers on all 3 subcohorts: glioma vs. PCNSL, high-grade glioma vs. PCNSL, and low-grade glioma vs. PCNSL.

**Table 2.** Results for different classifiers trained on the main cohort (Glioma vs. PCNSL) and two subcohorts (high-grade glioma vs. PCNSL and low-grade glioma vs. PCNSL).

Model	Strategy	Sensitivity	Specificity	Accuracy	ROC–AUC	F1-Score
Lymphomas vs. gliomas	Majority (glioma)	0	1.00	0.90	0.50	0
	Stratified	0	0.88	0.79	0.44	0
	Uniform	0.80	0.51	0.54	0.50	0.27
	XGBoost	0.6	1.0	0.96	0.99	0.75
Lymphomas vs. high-grade gliomas	Majority (glioma)	0	1.00	0.90	0.50	0
	Stratified	0.20	0.90	0.82	0.55	0.20
	Uniform	0.60	0.51	0.52	0.50	0.22
	XGBoost	0.6	0.95	0.909	0.91	0.6
Lymphomas vs. low-grade gliomas	Majority (glioma)	0	1.00	0.44	0.50	0
	Stratified	0.40	0.25	0.33	0.32	0.40
	Uniform	0.20	0.75	0.44	0.50	0.29
	XGBoost	0.8	1.0	0.89	0.95	0.89

### Glioma Versus PCNSL Classification Model

**Figure 3.**
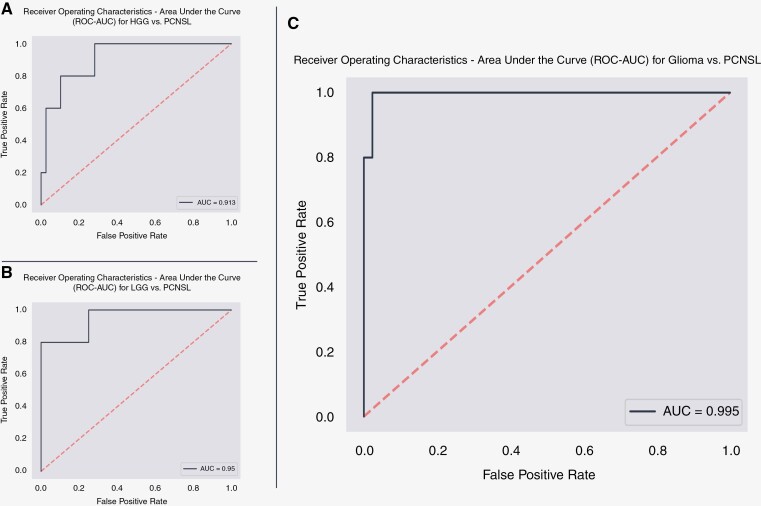
Receiver operating characteristics—area under the curve (ROC–AUC) for (a) high-grade glioma vs. PCNSL, (b) low-grade glioma vs. PCNSL, (c) glioma vs. PCNSL. An ROC curve, short for receiver operating characteristic curve, is a graphical representation illustrating the performance of a classification model across various classification thresholds. The curve is constructed by plotting two key parameters: true positive rate, false positive rate.

**Figure 4.**
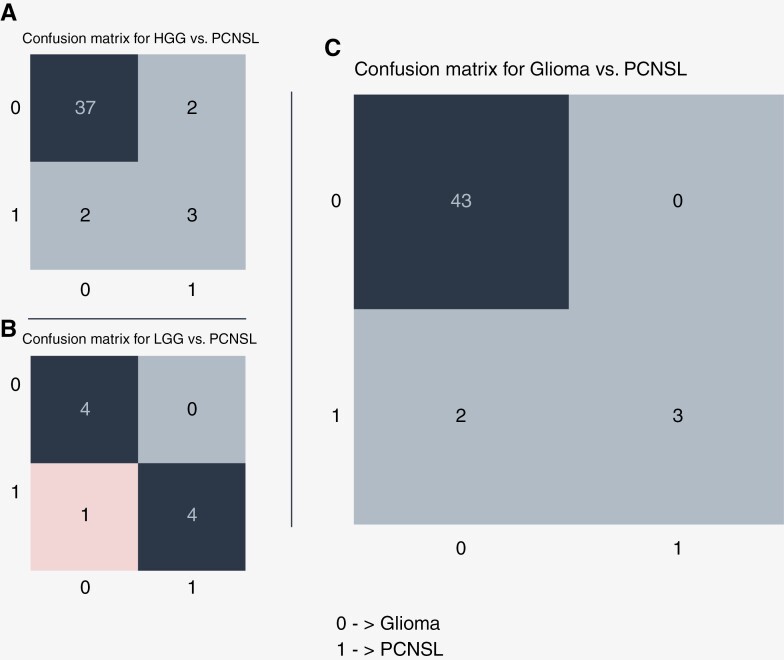
Confusion matrix for (a) high-grade glioma vs. PCNSL, (b) low-grade glioma vs. PCNSL, (c) glioma vs. PCNSL. The confusion matrix shows the number of correct and incorrect classifications from the models. As one can see, the model trained for LGG vs. PCNSL classifies all cases correctly except 1 PCNSL. The models for both glioma vs. PCNSL and HGG vs. PCNSL misclassified 2 PCNSL as gliomas. The glioma vs. PCNSL model was able to correctly classify all gliomas, whereas the HGG vs. PCNSL model misclassified 2 Gliomas as PCNSL.

The developed network referred to in this section is the model designed to distinguish between gliomas and PCNSLs accurately. The AUC for the developed network was 0.99 (Figure 3) on the test set, indicating that it could accurately differentiate between gliomas and PCNSLs. The sensitivity of the model, which measures the proportion of positive samples that were correctly classified, was 0.60. The model could correctly classify all gliomas in the test set. The specificity of the model, which measures the proportion of negative samples that were correctly classified, was 1.0. The model achieved an F1-score of 0.75 and an accuracy of 0.96. Figure 4C illustrates the confusion matrix on the test set for the model distinguishing gliomas from PCNSLs.

### High-Grade and Low-Grade Glioma Versus PCNSL Classification Models

In addition to the primary model for distinguishing between gliomas and PCNSLs, 2 separate models were also developed to differentiate between low-grade/high-grade gliomas and PCNSLs. These subtypes, high-grade and low-grade gliomas, are characterized by different levels of aggressiveness and corresponding image features. The model for distinguishing high-grade gliomas and PCNSLs achieved an AUC of 0.91, indicating that it could accurately differentiate between high-grade gliomas and PCNSLs. However, similar to the primary model, it reached a sensitivity of 0.60, a specificity of 0.95, and a low F1 score of 0.6. Similarly, the model for distinguishing low-grade gliomas and PCNSLs achieved an AUC of approximately 0.95, a sensitivity of 0.8, a specificity of 1.0, and an F1-score of 0.89. Figure 4A and B displays the confusion matrix on the test set for the model distinguishing HGG and LGG from PCNSLs, respectively.

## Discussion

The main aim of the current study was to differentiate PCNSLs from cerebral gliomas utilizing a simple routine MRI protocol in a fully automated workflow to enable seamless integration into the clinical routine. To raise the transferability, the MRI images in this study had different relaxation times, echo times, and slice thickness from day-to-day clinical routine scans from 1.5 T and 3 T MRI scanners.

While primary brain tumors have been well studied, and several criteria have been established for classifying different grades and types of brain tumors, the correct diagnosis of PCNSL is still challenging due to its complex heterogeneity.^39^ Hence, after the successful implementation of radiomics-based analysis for primary brain tumors, the differentiation of PCNSL from glioblastoma has been the focus of multiple studies.^6,7,18,19,21–23,40,41^

All these studies had strict requirements for MRI scans with different scanners. For this study, the scanner parameters were kept similar to those used in daily routines. For example, the relaxation time, the echo time, slice thickness, etc., was not fixed like in many studies mentioned above. This was done to consider that none of such variables are set in the day-to-day clinical routine. These variables depend on the radiologist and the consulting doctor and differ from hospital to hospital. Hence, training an algorithm based on MRIs taken with fixed parameters would force everyone to acquire MRI scans with those parameters or yield unsatisfactory results. The parameters used in the current study are mentioned in Supplementary Appendices B–D. Hence, the essential purpose of this study was to overcome these issues and make it easier to implement radiomics-based non-invasive tumor decoding in clinical practice.

Furthermore, most of these studies differentiated either high-grade gliomas or glioblastomas from PCNSLs. Liao et al.^20^ discovered that the difference in DSC perfusion MRI characteristics between PCNSLs and high-grade gliomas is determined by their different vascularity and different patterns of contrast agent leakage. This difference may help to distinguish between PCNSLs and high-grade gliomas, which sometimes may have a similar conventional MR imaging appearance. The studies mentioned above focused exclusively on differentiating glioblastomas from PCNSLs. However, other gliomas can also be confused with PCNSLs.

Furthermore, the new WHO classification^42^ has changed the definitions and classification of gliomas. Some LGG exhibits contrast-enhancing (CE); however, a third of HGG shows no contrast enhancement on baseline neuroimaging.^43–46^ At the same time, the treatment strategies for LGGs vary from other types of gliomas.^43^ Hence, it is necessary to differentiate all types of gliomas from PCNSLs.

Some of these studies used a manual,^7,18,19,21,22,40,41^ whereas some used a semi-automatic segmentation^6,23^ approach, the results of which are challenging to reproduce. The first step in this automated pipeline (workflow) was to strip down the skull from the MRI images to anonymize the data. This was achieved by using the HD-BET algorithm.^26^ For segmentation, an automatic approach was taken for this study to ensure reproducibility. HD-GLIO^24,25^ was integrated into the workflow by preprocessing the images according to the network requirements. Sørenson et al.^47^ evaluated the algorithm and achieved a mean Sørensen–Dice coefficient of 0.79 for non-enhancing (NE) and 0.86 for contrast-enhancing (CE) lesions. When Bouget et al. compared the Sørensen–Dice coefficient and segmentation time of the nnU-net (HD-GLIO) and the Attention-gated U-Net (AGU-net), the nnU-net fared better.^48^ Hence, HD-GLIO was chosen as the segmentation network of choice for this study. Due to the lack of T2-weighted MRI, the FLAIR sequence was passed twice to the network as input. To determine whether this change significantly affected the segmentations, experts manually validated the generated segmentations with the segmentations developed. The validation on 213 manually segmented glioma patients resulted in a mean Sørensen–Dice coefficient of 0.82 ± 0.13, whereas PCNSLs segmentations for 24 manually segmented patients yielded a mean Sørensen–Dice coefficient of 0.80 ± 0.19.

The segmentation and MRI images were then passed on further for feature extraction and selection. A hyperparameter search was also performed using OPTUNA for the XGBoost classifier, which provided an AUC of 0.99 on the test set, with an F1 score of 0.75. P. Alcaide-Leon et al.^49^ included 106 patients (71 with high-grade gliomas and 35 with PCNSLs) scanned in a 1.5 or 3T MR scanner. The study was based on a non-contrast T1-weighted sequence and a contrast-enhanced T1-weighted sequence scanned with fixed parameters for all patients. The study used a Support Vector Machine classifier instead of an XGBoost classifier, which resulted in an AUC of 0.88, which is lower than the results obtained in the current study. Compared to our study, manual segmentations were performed, which impedes transferability and clinical use; furthermore, low-grade gliomas were excluded from the analysis, in contrast to ours. Most of the literature focuses on differentiating glioblastomas from PCNSLs; hence, it becomes difficult to compare the results directly. However, these results are better than those obtained using texture analysis of T1-weighted images.^49^

Two more networks were trained to differentiate high-grade gliomas from PCNSLs and low-grade gliomas from PCNSLs. The network followed the same pipeline and achieved an AUC of 0.91 (F1 score of 0.6) for high-grade gliomas vs. PCNSLs and 0.95 (F1 score of 0.89) for low-grade gliomas vs. PCNSLs. As mentioned above, most available literature concentrates on differentiating PCNSL from glioblastoma. Some of these studies used radiomics features, whereas some used a combination of imaging and clinical features. The findings of this study closely align with, and in certain aspects surpass, those of the previously referenced investigations.

In conjunction with radiomic methodologies, ongoing investigations encompass liquid biopsy techniques involving the analysis of circulating tumor DNA in cerebrospinal fluid and plasma, offering potential non-invasive avenues for tumor diagnosis.^50^ It can be used in different areas, such as diagnostics, identifying potential therapeutic targets, tumor relapse, and many more. In future studies, it would be very interesting to combine liquid biopsy with radiomics-based workflows for either detecting tumors, survival analysis or for therapy response.

However, this study also had some limitations. The data was monocentric; therefore, future research should aim for multicentric or external data validation. Additionally, the dataset was highly imbalanced, affecting the model's classification capabilities. In view of the limited number of PCNSL patients, this study should be considered as a proof-of-concept and requires further evaluation with the help of prospective, preferably multicentric data. Nonetheless, the developed machine learning models performed significantly better than dummy classifiers using the same datasets. Vitally, the misclassification of glioma as a PCNSL is of much more significant impact, as higher-grade cerebral gliomas usually undergo biopsy, whereas PCNSLs are typically treated non-invasively.

## Conclusions

In summary, this study introduces a comprehensive automated workflow for discriminating between PCNSLs and cerebral gliomas, marking a significant stride toward realizing an entirely automated virtual biopsy process for cerebral lesions. The models developed exhibit robustness when confronted with variations in scanner parameters, encompassing field strength, imaging parameters, and diverse scanner systems. This study serves as a pilot investigation aimed at establishing a fully automated MRI-based workflow incorporating radiomics, with the purpose of discriminating between PCNSLs and cerebral gliomas. Validating the results in a prospective multicenter setting with a higher number of PCNSL patients is required before clinical implementation.

## Supplementary Material

vdae022_suppl_Supplementary_Appendix

## Data Availability

The used dataset within this study is available from the corresponding author upon reasonable request. The dataset would be shared as a download link from the University’s cloud infrastructure.
