# Supplementary material for: Fully automated MR-based virtual biopsy of primary CNS lymphomas
Source: Neurooncol Adv. 2024 Mar 14;6(1):vdae022. doi: 10.1093/noajnl/vdae022 (PMC10956963; doi:10.1093/noajnl/vdae022)
Supplement: vdae022_suppl_Supplementary_Appendix [file vdae022_suppl_supplementary_appendix.docx]

# Appendix

Appendix A: Distribution of MRI images taken from different scanners in train and test sets.

| Scanner | Number of scans (train + test = total) |
| --- | --- |
| Aera | 58 + 17 = 75 |
| Skyra | 52 + 13 = 65 |
| Espree | 27 + 8 = 35 |
| Biograph_mMR | 19 + 4 = 23 |
| Avanto | 17 + 3 = 20 |
| Symphony | 9 + 1 = 10 |
| Sonata | 7 + 1 = 8 |
| Unknown | 3 + 1 = 4 |

Appendix B: Slice thickness from different scanners for train and test sets.

| Scanner | Set | Slice thickness (mean ± std) (mm) | | |
| --- | --- | --- | --- | --- |
|  | **Series** | **FLAIR** | **T1-pre-contrast** | **T1-post-contrast** |
| Aera | Train | 5.06 ± 0.89 | 5.08 ± 0.82 | 1.33 ± 1.21 |
|  | Test | 5.23 ± 0.43 | 5.23 ± 0.43 | 1.0 ± 0.0 |
| Skyra | Train | 4.75 ± 1.28 | 5.13 ± 0.34 | 1.30 ± 1.07 |
|  | Test | 4.92 ± 1.25 | 5.23 ± 0.43 | 1.0 ± 0.0 |
| Espree | Train | 5.77 ± 0.97 | 5.96 ± 0.19 | 1.74 ± 1.81 |
|  | Test | 6.0 ± 0.0 | 6.0 ± 0.0 | 2.87 ± 2.58 |
| Biograph_mMr | Train | 4.57 ± 1.64 | 5.21 ± 0.42 | 1.0 ± 0.0 |
|  | Test | 5.0 ± 0.0 | 5.0 ± 0.0 | 1.0 ± 0.0 |
| Avanto | Train | 5.64 ± 0.49 | 5.64 ± 0.49 | 1.0 ± 0.0 |
|  | Test | 5.50 ± 0.57 | 5.50 ± 0.57 | 1.10 ± 0.20 |
| Symphony | Train | 6.0 ± 0.0 | 6.0 ± 0.0 | 1.66 ± 1.80 |
|  | Test | 6.0 ± 0.0 | 6.0 ± 0.0 | 1.0 ± 0.0 |
| Sonata | Train | 6.0 ± 0.0 | 6.0 ± 0.0 | 1.71 ± 1.88 |
|  | Test | 6.0 ± 0.0 | 6.0 ± 0.0 | 1.0 ± 0.0 |

Appendix C: Echo time from different scanners for train and test sets.

| Scanner | Set | Echo Time (mean ± std) (ms) | | |
| --- | --- | --- | --- | --- |
|  | **Series** | **FLAIR** | **T1-pre-contrast** | **T1-post-contrast** |
| Aera | Train | 112.94 ± 42.33 | 7.93 ± 2.25 | 3.67 ± 2.11 |
|  | Test | 104.17 ± 2.32 | 8.06 ± 2.30 | 3.11 ± 0.09 |
| Skyra | Train | 131.57 ± 87.76 | 8.35 ± 1.35 | 2.70 ± 1.83 |
|  | Test | 124.15 ± 83.40 | 8.17 ± 1.49 | 2.20 ± 0.05 |
| Espree | Train | 120.22 ± 46.93 | 12.07 ± 2.92 | 5.32 ± 4.96 |
|  | Test | 111.00 ± 0.00 | 14.0 ± 0.0 | 8.40 ± 7.12 |
| Biograph_mMr | Train | 141.47 ± 112.64 | 11.81 ± 2.56 | 3.01 ± 0.21 |
|  | Test | 94.0 ± 0.0 | 13.0 ± 0.0 | 2.86 ± 0.0 |
| Avanto | Train | 109.0 ± 0.0 | 8.62 ± 1.17 | 3.11 ± 0.07 |
|  | Test | 109.0 ± 0.0 | 8.92 ± 0.76 | 3.14 ± 0.11 |
| Symphony | Train | 115.0 ± 0.0 | 8.10 ± 0.0 | 6.83 ± 5.76 |
|  | Test | 115.0 ± 0.0 | 8.10 ± 0.0 | 3.93 ± 0.0 |
| Sonata | Train | 108.0 ± 0.0 | 8.10 ± 0.0 | 5.79 ± 4.94 |
|  | Test | 108.0 ± 0.0 | 8.10 ± 0.0 | 3.93 ± 0.0 |

Appendix D: Repetition time from different scanners for train and test sets.

| Scanner | Set | Repetition Time (mean ± std) (ms) | | |
| --- | --- | --- | --- | --- |
|  | **Series** | **FLAIR** | **T1-pre-contrast** | **T1-post-contrast** |
| Aera | Train | 8862.06 ± 736.23 | 457.43 ± 218.32 | 1830.53 ± 370.22 |
|  | Test | 9000.0 ± 0.0 | 437.64 ± 117.23 | 1928.23 ± 23.24 |
| Skyra | Train | 8615.38 ± 1190.71 | 1805.30 ± 529.61 | 1792.34 ± 380.93 |
|  | Test | 8692.30 ± 1109.40 | 1751.38 ± 606.86 | 1892.30 ± 34.19 |
| Espree | Train | 8888.88 ± 577.35 | 407.78 ± 52.12 | 1720.66 ± 561.17 |
|  | Test | 9000.0 ± 0.0 | 443.75 ± 25.46 | 1345.87 ± 798.03 |
| Biograph_mMr | Train | 8368.42 ± 1498.53 | 2260.10 ± 564.42 | 2020.0 ± 81.24 |
|  | Test | 9000.0 ± 0.0 | 2080.0 ± 0.0 | 2070.0 ± 0.0 |
| Avanto | Train | 9000.0 ± 0.0 | 430.52 ± 95.13 | 1980.58 ± 69.59 |
|  | Test | 9000.0 ± 0.0 | 500.25 ± 108.43 | 1987.50 ± 15.0 |
| Symphony | Train | 9000.0 ± 0.0 | 391.11 ± 17.63 | 1670.77 ± 643.74 |
|  | Test | 9000.0 ± 0.0 | 360.0 ± 0.0 | 2030.0 ± 0.0 |
| Sonata | Train | 9000.0 ± 0.0 | 394.28 ± 15.11 | 1814.28 ± 544.26 |
|  | Test | 9000.0 ± 0.0 | 393.33 ± 11.54 | 1960.00 ± 103.92 |

Appendix E: Technical project-related terminologies and their description.

| Terminology | Description |
| --- | --- |
| BorutaPy | BorutaPy is an implementation of the Boruta algorithm in Python. It is a widely used feature selection method that selects all relevant features pertaining to the outcome of the classifier^1,2^. |
| HD-BET | HD-BET is an automated brain extraction tool developed as a joint project between the Department of Neuroradiology at the Heidelberg University Hospital and the Division of Medical Image Computing at the German Cancer Research Center (DKFZ)^3,4^. |
| HD-GLIO | HD-GLIO is a brain tumor segmentation tool. It was a joint project between the Department of Neuroradiology at the Heidelberg University Hospital, Germany and the Division of Medical Image Computing at the German Cancer Research Center (DKFZ) Heidelberg, Germany^5,6^. |
| Sørensen–Dice coefficient | The Dice similarity coefficient, also known as the Sørensen–Dice index or simply Dice coefficient, is a statistical tool which measures the similarity between two sets of data^7^. |
| XGBoost | XGBoost is a scalable machine learning system for tree boosting algorithm^8,9^. |

Appendix F: Distribution of the total cohort of the respective genetic parameters. A positive label is defined as having an IDH1/2 mutation, an ATRX expression loss, a 1p19q co-deletion, and a positive MGMT methylation status. For the WHO label, LGG is defined as positive, and HGG as negative.

| Label | Distribution (positive / negative) |
| --- | --- |
| MGMT | 164 (81/83) |
| IDH1/2 | 145 (34/111) |
| 1p19q | 30 (5/25) |
| ATRX | 67 (13/54) |
| WHO | 216 (30/186) |
| PCNSL | 24 |

# Supplementary References

1. Kursa MB, Rudnicki WR. Feature Selection with the **Boruta** Package. *J Stat Soft*. 2010;36(11). doi:10.18637/jss.v036.i11

2. BorutaPy: All relevant feature selection method. Daniel Homola. Published May 8, 2015. Accessed October 20, 2021. https://danielhomola.com/feature%20selection/phd/borutapy-an-all-relevant-feature-selection-method/

3. MIC-DKFZ/HD-BET. Published online January 9, 2024. Accessed January 13, 2024. https://github.com/MIC-DKFZ/HD-BET

4. Isensee F, Schell M, Pflueger I, et al. Automated brain extraction of multisequence MRI using artificial neural networks. *Hum Brain Mapp*. 2019;40(17):4952-4964. doi:10.1002/hbm.24750

5. Isensee F, Jäger PF, Kohl SAA, Petersen J, Maier-Hein KH. Automated Design of Deep Learning Methods for Biomedical Image Segmentation. *Nat Methods*. 2021;18(2):203-211. doi:10.1038/s41592-020-01008-z

6. NeuroAI-HD/HD-GLIO. Published online December 13, 2023. Accessed January 13, 2024. https://github.com/NeuroAI-HD/HD-GLIO

7. Moore CM. Dice similarity coefficient | Radiology Reference Article | Radiopaedia.org. Radiopaedia. doi:10.53347/rID-75056

8. Chen T, Guestrin C. XGBoost: A Scalable Tree Boosting System. In: *Proceedings of the 22nd ACM SIGKDD International Conference on Knowledge Discovery and Data Mining*. ACM; 2016:785-794. doi:10.1145/2939672.2939785

9. XGBoost Documentation — xgboost 2.0.3 documentation. Accessed January 13, 2024. https://xgboost.readthedocs.io/en/stable/
